# Role of hypoxia in cancer therapy by regulating the tumor microenvironment

**DOI:** 10.1186/s12943-019-1089-9

**Published:** 2019-11-11

**Authors:** Xinming Jing, Fengming Yang, Chuchu Shao, Ke Wei, Mengyan Xie, Hua Shen, Yongqian Shu

**Affiliations:** 1 Department of Oncology, The Affiliated Sir Run Run Hospital of Nanjing Medical University, Nanjing, China; 2 Department of Oncology, The First Affiliated Hospital of Nanjing Medical University, Nanjing, China; 3 Department of Thoracic surgery, The First Affiliated Hospital of Nanjing Medical University, Nanjing, China

**Keywords:** Cancer therapy, Chemotherapy, Drug resistance, Hypoxia, Tumor microenvironment

## Abstract

**Aim:**

Clinical resistance is a complex phenomenon in major human cancers involving multifactorial mechanisms, and hypoxia is one of the key components that affect the cellular expression program and lead to therapy resistance. The present study aimed to summarize the role of hypoxia in cancer therapy by regulating the tumor microenvironment (TME) and to highlight the potential of hypoxia-targeted therapy.

**Methods:**

Relevant published studies were retrieved from PubMed, Web of Science, and Embase using keywords such as hypoxia, cancer therapy, resistance, TME, cancer, apoptosis, DNA damage, autophagy, p53, and other similar terms.

**Results:**

Recent studies have shown that hypoxia is associated with poor prognosis in patients by regulating the TME. It confers resistance to conventional therapies through a number of signaling pathways in apoptosis, autophagy, DNA damage, mitochondrial activity, p53, and drug efflux.

**Conclusion:**

Hypoxia targeting might be relevant to overcome hypoxia-associated resistance in cancer treatment.

## Introduction

Cancer is a major public health problem worldwide with few effective treatment choices, poor prognosis, and high mortality rates [1]. The antitumor treatments are based on chemotherapy, radiation therapy, and targeted therapy. However, acquired resistance has become a serious challenge for anticancer therapies. Cancer cells acquire resistance through a variety of mechanisms and signaling involving both intrinsic and extrinsic factors. In the majority of patients with end-stage cancer, the primary cause for treatment failure is resistance to cancer therapy. Either primary or acquired drug resistance particularly represents a significant impediment in clinical oncology. Therefore, studying the mechanisms of drug resistance is important in parallel with the development of the drug itself. Both pharmacological factors, including inadequate drug concentration at the tumor site, and cellular factors can contribute to clinical resistance [2]. The precise mechanisms of drug resistance in tumors are complex and multifactorial, but they can be grouped into three categories: insufficiency of pharmacokinetic properties, intrinsic factors of tumor cells, and external conditions of tumor cells in the tumor microenvironment (TME) [3].

Mounting studies have confirmed that the TME promotes cancer progression in many aspects, especially therapeutic resistance. The TME decreases drug penetration and confers the advantage of proliferation and antiapoptosis to surviving cells, facilitating resistance and common modifications in disease morphology [4, 5]. Soluble factors secreted by tumor or stromal cells are rich in the TME and contribute to abnormal proliferation, angiogenesis, metastasis, and drug resistance [6]. As the rapid and uncontrolled proliferation of tumors limits the availability of oxygen, insufficient blood supply, or hypoxia is a typical microenvironment feature in nearly all solid tumors [7]. Oxygen is essential for energy metabolism to drive cellular bioenergetics. The rapid proliferation of tumors outgrows their surrounding vasculature, resulting in a drop of normal oxygen levels of 2–9% to hypoxic levels of less than 2%. Regions with low oxygen levels are generally termed as hypoxic regions. Extensive reviews have been reported about the clinical significance of hypoxia in cancer therapy [8–10]. Diminished oxygen availability (hypoxia), as a hallmark of the TME, presents in the majority of tumors, arising from an imbalance between increased oxygen consumption and inadequate oxygen supply. Although the rapid proliferation of tumors can stimulate the growth of new vasculature and the tumor-induced angiogenesis leads to the unorganized growth of vasculature, the precisely distributed vasculature in normal tissues contributes to the delivery of oxygenated blood. The irregular distribution of tumor vasculature caused by persistent hypoxic conditions can result in an increase in the distance between the capillaries, exceeding the capacity of oxygen to diffuse [11, 12]. Such chronic hypoxia or diffusion-restricted hypoxia causes the necrosis of tumor cells within the 180-μm periphery of blood vessels. However, the current anticancer strategies target only tumor cells around the blood vessels rather than those in poorly perfused regions [13, 14]. The presence of hypoxic regions is one of the independent prognostic factors for human cancer. Tumor cells, while adapting to hypoxia, lead to more aggressive and therapeutically resistant tumor phenotypes. Hypoxia induces changes in gene expression and subsequent proteomic changes that have many important effects on various cellular and physiological functions, ultimately limiting patient prognosis [15]. For example, slowly dividing cells in hypoxic regions can escape most of the cytotoxic drugs that target rapidly dividing cells, and cancer stem cells may also be present in poorly hypoxic regions ensuring epithelial-to-mesenchymal transition (EMT) [16].

Hypoxia generates intratumoral oxygen gradients, contributing to the plasticity and heterogeneity of tumors and promoting a more aggressive and metastatic phenotype. In this process, the increased expression of hypoxia-inducible factor 1α (HIF-1α) is a pivotal hallmark. HIFs play a central role in cellular mechanisms triggered in response to hypoxia [17, 18]. HIF is a heterodimer composed of two basic helix-loop-helix proteins of the Per-ARNT-Sim (PAS) family: an oxygen-sensitive α-subunit and a constitutively expressed β-subunit [19]. Three HIF-α isoforms have been identified in mammals. Compared with HIF-1, a transcriptional nucleoprotein with a wide range of target genes, HIF-2 seems to be more restricted in expression in the tissue, and less is known about HIF-3 [20]. The oxygen status can regulate the stability of HIF-α family proteins. Under normoxic conditions, two critical proline residues in HIF-α subunits are subject to hydroxylation within their oxygen-dependent degradation domain by enzymes called HIF prolyl hydroxylase domain family proteins (PHDs), which use O_2_, ferrous iron, and α-ketoglutarate as substrates. PHDs are HIF-preserved hydroxylases found in mammals, with three subtypes PHD1, PHD2, and PHD3, as regulators of HIF-1α oxygen sensors to participate in the degradation of HIF-1α. PHD2 keeps HIF-1α at a stable low level in an anoxic environment as the main rate-limiting enzyme, and its activity is controlled mainly by the intracellular oxygen concentration. Then, the von Hippel–Lindau tumor suppressor protein (pVHL) interacts with HIF-α as a result of hydroxylation and recruits an E3 ubiquitin ligase complex, resulting in ubiquitination and subsequent proteasomal degradation of HIF-α. Under hypoxic conditions, the inhibitory hydroxylation of HIF-α is reduced, leading to the stability and translocation of HIF-α to the nucleus, where it heterodimerizes with HIF-β [21]. The HIF-α/β dimer binds with the transcriptional coactivator p300/CBP and hypoxia response element to induce the expression of the HIF target gene located in the promoter region [22, 23]. HIFs play a distinct role in tumorigenesis, and immunohistochemical analyses show that HIF-1α and HIF-2 α are overexpressed in the majority of human cancers. Especially in recent years, more attention has been paid to HIF-1 and drug resistance in a wide spectrum of neoplastic cells [24, 25].

**Fig. 1:**
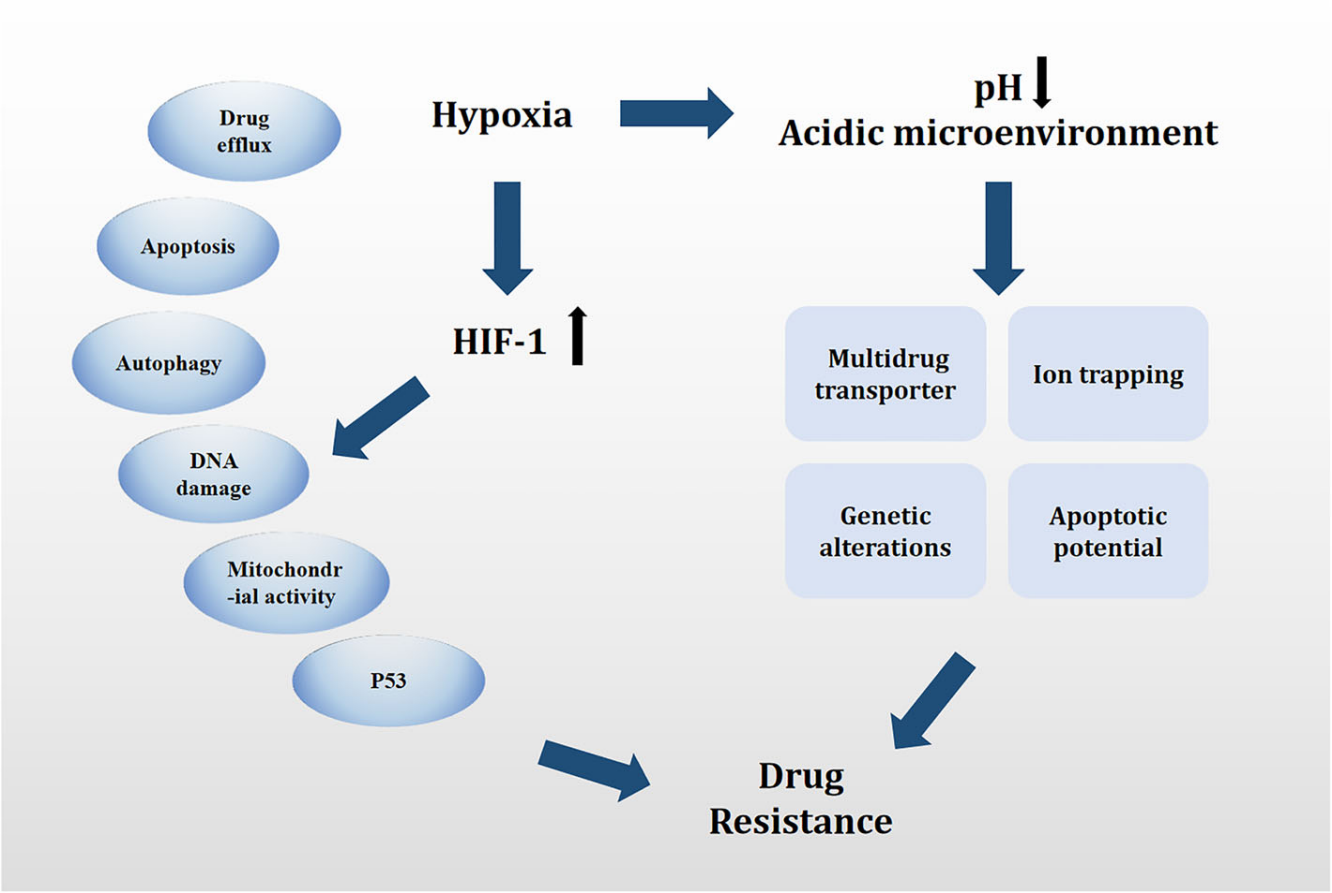
Summary of mechanisms and pathways of HIF-mediated drug therapy failure. HIF-1 confers resistance to conventional therapies through a number of signaling pathways in apoptosis, autophagy, DNA damage, mitochondrial activity, p53, and drug efflux. In addition, hypoxia results in a decrease in pH and creates an acidic TME. Mechanisms by which the tumor acidic microenvironment leads to MDR, including a decreased concentration of the drug caused by “ion trapping,” reduced apoptotic potential, genetic alterations (such as p53 mutations), and elevated activity of a multidrug transporter p-glycoprotein (P-gp)

This study aimed to focus on the cause of therapy resistance from the perspective of tumor cell adaptation to a hypoxic microenvironment, particularly discussing the capacity of oxygen-regulated transcription factor HIF-1 in modifying cancer sensitivity to therapeutic agents. Specifically, it aimed to provide an overview of the effect of hypoxia and HIFs on anticancer drug resistance, highlighting the multifaceted interaction of HIFs with apoptosis, autophagy, DNA damage, mitochondrial activity, and p53 in the failure of HIF-mediated therapy (summarized in Fig. 1 and Table 1). Finally, the study worked toward providing a comprehensive understanding of hypoxia-mediated molecular signaling pathways and a new sight for cancer therapy.

**Table 1:** Overview of HIF-1-mediated mechanisms in drug resistance

Resistance phenotype	Cancer/Cell type	Resistant chemotherapy drug	Molecular basis	Reference
Overexpression of drug efflux proteins	Colon cancer cells	5-Fluorouracil	MDR1/P-gp	[26]
Overexpression of drug efflux proteins	Ovarian carcinoma cells	Estramustine	ABCA2	[27]
Overexpression of drug efflux proteins	Lung adenocarcinoma cells	Adriamycin	P-gp	[28]
Apoptosis inhibition	Breast cancer cells	Paclitaxel	Caspases 3, 8, 10, and Bak	[29]
Apoptosis inhibition	Colon cancer cells	Etoposide and oxaliplatin	Bid and Bax	[30]
Apoptosis inhibition	Gastric cancer cells	5-Fluorouracil and cisplatin	p53 and NF-kB	[31]
Apoptosis inhibition	Human melanoma cells	Not mentioned	P53 and TRP2	[88]
Autophagy induction	HeLa cells	N-(4-Hydroxypheny) retinamide (4-HPR)	Beclin1	[32]
Autophagy induction	Gastric cancer cells	Vincristine	miR-23b-3p, ATG12, and HMGB2	[33]
Autophagy induction	Colon cancer cells	Cryptotanshinone Dihydrotanshinone	p53	[34]
DNA damage inhibition	Mouse embryonic fibroblasts	Etoposide	DNA–PKcs and Ku80	[35]
DNA damage inhibition	Breast and liver cancer cells	Taxol and etoposide	TMEM45A	[36]
Mitochondrial activity	Human leukemia cell line (HL-60) human lymphoma cell line (Raji)	Doxorubicin and ara-c	BAD	[37]
Mitochondrial activity	Renal carcinoma cells	Not mentioned	VHL	[38]
Mitochondrial activity	Oral squamous cell carcinoma cells	5-Fluorouracil and cisplatin	Cytochrome, Akt, and ERK	[39]
P53	Non-small-cell lung cancer cells	Cisplatin	HIF-1α and BAX	[40]

### Hypoxia and TME

Tumor cells develop new blood vessels to adapt to low levels of oxygen and nutrients, which is called de novo angiogenesis. However, such newly formed blood vessels are leaky because of their discontinuous endothelium, along with the obstruction of lymphatic drainage, producing vascular hyperpermeability and enhanced permeation [41]. Hence, hypoxia causes vascular leakage and abnormal lymphatic drainage in the tumor, leading to an increase in interstitial fluid pressure [42]. TME refers to the local biological environment in which solid tumors are located, including cancer cells and nearby stromal cells. Cancer cells select normal host cells, such as fibroblasts, various immune cells, and blood and lymphatic cells, which are embedded in tightly packed extracellular matrices. The secondary formation of harsh metabolic and physical microenvironments results in an imbalance of positive and negative regulators of processes in activating and deregulating angiogenesis, desmoplasia, and inflammation. Most tumors have hypoxic regions. The development of an abnormal vasculature and a hypoxic microenvironment promotes abnormal angiogenesis, desmoplasia, and inflammation, all of which contribute to tumor progression and therapeutic resistance [43, 44]. HIF-1α mediates hypoxia-induced signaling, which plays a role in multiple steps of the transfer cascade [23]. Tumor metastasis is the migration of cancer cells from the primary tumor in the form of single cells or multiple cell clusters to distant sites in the body. Studies have shown that cancer-associated fibroblasts (CAFs) and myeloid cells assist in the process of tumor metastasis [45]. In a hypoxic environment, activated HIF-1α increases the activity of Snail and Twist, two transcription factors that reduce E-cadherin expression and promote EMT. More interestingly, EMT-related signaling is not required for the metastatic process, but it promotes invasion, aging, cancer stem cell–like phenotype, and resistance to chemotherapy [46]. HIF-1α can also intervene in the expression of enzymes that polymerize and regulate the alignment of collagen fibers and activity of integrins to promote cancer migration [23]. Finally, as already mentioned, hypoxia fosters leaky and compressed blood and lymphatic vessels mediated by HIFs such as angiopoietin-2, vascular endothelial growth factor (VEGF), and angiopoietin-like 4, facilitating the passage of metastatic cancer cells through the vessel wall [47].

In addition, the anoxic microenvironment is beneficial for glycolysis and lactic acid production by key enzymes of glycolysis and lactate dehydrogenase A (LDH-A); the excess production of lactic acid results in acidic pH. Moreover, HIF can reversely convert carbon dioxide and water produced by the activation of carbonic anhydrase IX or XII into HCO_3_^−^, which diffuses out of the cell membrane, resulting in excess HCO_3_^−^ in the TME and a decrease in pH [9]. A large number of studies have concluded that the decreased intracellular pH of endosomes and lysosomes in tumor cells may assist in metastasis by activating proteases [42, 48]. In fact, alterations in extracellular pH induce drug resistance by inhibiting cellular and humoral immune functions because acidic pH predominates at loci of inflammation and other immunologically active sites. Investigations have shown that reduced pH inhibits mainly the chemotaxis, respiratory activity, and bactericidal ability of polymorphonuclear leukocytes. Moreover, impaired cytotoxicity and proliferation of lymphocytes at acidic pH have been reported. Similarly, the decreased lysis of various tumor cell lines by cytotoxic T lymphocytes at acidic extracellular pH has been demonstrated. The neutralization of T-cell effect or function and tumor acidity can improve the response to immunotherapy [49]. Studies on macrophages and eosinophils have suggested acid-induced activation of complement proteins and alternative complement pathway, coupled with increased binding of antibodies to leukocytes at a lower pH. The level of reactive oxygen species (ROS) has been shown to be increased in cancer cells exposed to hypoxia [50]. The reduction in oxygen utilization decreases the passage of electrons through the mitochondrial complex by the electron transport chain (ETC), allowing electrons to leak from the ETC, thus leading to the overproduction of ROS [51]. Moreover, the excessive production of ROS alters genomic stability and impairs DNA repair pathways [52]. ROS can cause cell survival or apoptosis via a mechanism referred to as oxidative stress, thus resulting in enhanced cytotoxicity and apoptosis [53]. At high concentrations (10–30 μm), ROS can damage cellular biomolecules, such as proteins, DNA, and RNA, and cause mutations that promote cancer in normal cells or multidrug resistance (MDR) in cancer cells [54]. However, most cancer cells still survive under internal oxidative stress, hence avoiding apoptosis and developing resistance to chemotherapy. Exposure to elevated levels of ROS can lead to cancer cell resistance by the activation of redox-sensitive transcription factors such as NF-κB, nuclear factor (erythroid-derived 2)-like factor 2 (Nrf2), c-Jun, and HIF-1α [55]. Subsequently, the activation of these genes enhances the activation of the antioxidant system and promotes the expression of cell survival proteins. In addition, ROS facilitate the transition from apoptosis to autophagy in methotrexate-resistant choriocarcinoma jeg-3 cells, enabling the survival of cells to methotrexate [56]. ROS can also stimulate the differentiation of cancer stem cells, thus promoting EMT and inducing metabolic reprogramming involved in the resistance of cancer cells.

Hypoxic stress causes immunosuppression by controlling angiogenesis and favoring immune suppression and tumor resistance. Macrophages constitute a principal component of the immune infiltrate in solid tumors by differentiating into tumor-associated macrophages (TAMs), which have been found to be preferentially located in tumor hypoxic areas [57]. Tumor-derived cytokines are able to convert TAMs into polarized type 2, or M2, macrophages with more immunosuppressive activities, resulting in tumor progression. Myeloid-derived suppressor cells (MDSCs) can directly promote immune tolerance [58]. In hypoxic zones, HIF-1 directly regulates the function and differentiation of MDSCs, and such tumor-derived MDSCs are more immunosuppressive compared with splenic MDSCs. A recent study showed the upregulation of the expression of programmed death-ligand 1 (PD-L1) under hypoxia [59]. Further evidence supports that HIF-1 is a major regulator of PD-L1 mRNA and protein expression. HIF-1 regulates the expression of PD-L1 by binding directly to a hypoxia response element in the PD-L1 proximal promoter [58]. The originally elevated immunosuppressive function of tumor-derived MDSCs under hypoxia was found to be abrogated following PD-L1 blockade. Along with PD-L1 blockade, the hypoxia-mediated upregulation of IL-6 and IL-10 in MDSCs was significantly attenuated [60]. At present, immunotherapeutic strategies triggering antitumor immunity are not effective because of diverse mechanisms of tumor escape from immunosurveillance. The antibody blockade of the T-cell immune checkpoint receptors PD-1 and CTLA-4 was poor in some tumors because T cells were sparse or absent in the TME; the hypoxia-driven modulation of T-cell exclusion and apoptosis help maintain this state. T cells can enter hypoxic tumors. The hypoxia-mediated acidification of the extracellular milieu blocks the capacity of T cells to expand or perform cytotoxic effector functions. Taken together, tumor hypoxia predicts poor outcomes across all cancers [61]. It is clear that hypoxia plays a prominent role in establishing and maintaining tumor immune privilege or immunotherapy. It is conceivable that given the central role of hypoxia in the regulation of tumor progression and immune suppression, it might be considered in new combined cancer therapies.

### Hypoxia and cancer therapy

In recent years, the contribution of hypoxia to tumor therapy, especially drug resistance, has been observed in a wide range of neoplastic cells [62–65]. Roland Wenger and colleagues first studied the effects of hypoxia on the proliferation of mouse embryonic fibroblasts. When HIF-1 is inactivated, the inhibitory effect of carboplatin and etoposide on cell proliferation is significantly enhanced [66]. The HIF-1α protein is overexpressed in various common solid malignant tumors, including breast, colon, gastric, lung, skin, ovarian, pancreatic, prostate, and renal carcinomas compared with their respective normal tissues [67–69]. For example, the significantly increased expression level of HIF in pancreatic cancer is primarily used to evaluate the prognosis of patients clinically. A large number of studies have shown that HIF-1α can be used as a marker of the survival rate of pancreatic cancer [70–72]. The expression level of HIF-1α, VEGF, and glucose transporter protein 1 was found to be increased significantly using the immunohistochemical technique in 58 patients with pancreatic cancer and 20 normal human tissue samples. The Cox regression analysis revealed that HIF-1α was an independent marker for evaluating the prognosis and survival of patients with pancreatic cancer. Further comparison of HIF-1α levels in tissues of patients with short-term survival (< 6 months) and long-term survival (6–60 months) suggested that HIF-1α was a prognostic marker with the specificity of 87.1% and sensitivity of 55.6% [72]. It reflected that HIF-1α could be used to evaluate the survival time of patients with pancreatic cancer after diagnosis. Hence, it was concluded that the expression of HIF-1α was linked to the clinicopathological features and affected the survival of patients. Moreover, quantitative data emphasized the significance of HIF-1α in the prognostic evaluation of pancreatic cancer.

Hypoxia leads to a decreased pH in the TME. Hypoxia induced by the acidic TME leads to MDR via various mechanisms, including a decreased concentration of the drug caused by “ion trapping,” reduced apoptotic potential, genetic alterations (such as p53 mutations), and elevated activity of a multidrug transporter p-glycoprotein (P-gp). Uncharged small molecules could easily diffuse through the phospholipid portion of the cell membrane, whereas charged molecules could not, providing a condition for the acidic extracellular environment of the solid tumor to become weakly alkaline. This phenomenon is called “ion trapping [73].” However, some chemotherapeutic drugs currently used in clinical practice are pH dependent in terms of their intracellular targets. Hence, changes in the intracellular pH gradient resulted in decreased drug accumulation in tumor cells, thereby greatly reducing the efficacy of chemotherapeutic drugs and eventually leading to drug resistance. Some studies demonstrated that the efficacy of benzoate mustard in vivo was 2.3 times higher than that of doxorubicin. However, the efficacy of sodium bicarbonate in studying nitrogen mustard in vitro or in vivo drastically reduced after the alkalinization of the tumor environment. Melphalan, a slightly acidic chemotherapy drug approved for treating multiple myeloma and ovarian cancer, also showed consistent results with enhanced efficacy in preclinical and clinical trials on melanoma due to local hypoxia and a slightly acidic environment [74]. One of the main mechanisms of drug resistance was the expression of multidrug transport P-gp, which was encoded by the *MDR1* gene and resulted in the pumping out of cytotoxins such as doxorubicin and paclitaxel. Although its mRNA levels remained unchanged in the acidic environment, its activity increased, and this effect was doubled in low-oxygen environments. Studies showed no statistically significant difference in the expression level of P-gp after the treatment of A549 cells in the acidic medium, but the activity of P-gp was significantly enhanced and the peak appeared after 6 h. However, the cytotoxicity of daunomycin was significantly reduced and reversed under the synergistic effect of verapamil [75]. Otherwise, the p53-mutant cells were selected, causing p53-dependent apoptosis. This loss of apoptotic potential and a string of adaptive changes were likely driven by microenvironment-induced genomic instability and inhibition of DNA repair. Subsequently, data on the importance of hypoxia to the sensitivity of cancer cells under normoxic conditions are available. For example, in the hypoxic core of advanced solid tumors, a series of chain reactions caused by the high infiltration of immune cells enhanced the expression of the original gene, promoted tumor malignancy, and resulted in the emergence of drug resistance [76]. The use of cell-based targeted nanoparticles for effective therapy has been highlighted as a dual-mode treatment strategy to combat drug resistance and improve the efficacy of chemotherapy [77]. Therefore, hypoxia has been widely recognized as an active participant in tumor progression, affecting cell expression programs and therapeutic resistance. HIF-1, as the molecular basis, is commonly overexpressed in a majority of tumors, including breast, prostate, lung, and pancreatic carcinomas, besides head and neck cancer. The following section outlines general pathways and molecular mechanisms underlying the effect of HIF-1.

### Hypoxia-mediated overexpression of drug efflux proteins

The first proposed explanation is that HIF-1 can activate the multidrug resistance 1 (*MDR1*) gene in response to hypoxia. The quantitative microarray analysis of RNA revealed a sevenfold increase in MDR in epithelial cells exposed to hypoxia (pO2 20 Torr, 18 h); these findings were further confirmed at the mRNA and protein levels. The torr is the unit of pressure based on an absolute scale (one torr ≈133.322 Pa). MDR1 encodes for the membrane-resident P-gp, a member of ATP-binding cassette (ABC) transporter family, acting as a drug efflux pump to decrease the intracellular concentration of a series of chemotherapeutic drugs. The ABC transporters are known as a large family of integral membrane proteins, with at least 48 members in humans. Further, 12 of them have been recognized as putative drug transporters, including well-known P-gp encoded by the *ABCB1* gene, MDR-associated protein 1 (MRP1, encoded by the *ABCC1* gene), and ABC subfamily G member2, also known as breast cancer resistance protein, which is encoded by the *ABCG2* gene [78, 79]. A study using a chemotherapeutic sensitivity assay and flow cytometry (FCM) to analyze the relationship between HIF-1 expression and sensitivity to chemotherapy revealed that HIF-1α inhibition reversed MDR in colon cancer cells via the downregulation of MDR1/P-gp [26]. Further analysis revealed that ABC2 was amplified and overexpressed in an estramustine (EM)-resistant human ovarian carcinoma cell line, and antisense-mediated downregulation of ABC2 expression sensitized the cell line to EM. Thus, the overexpression of ABC2 contributed to EM resistance by serving as an efflux pump for chemotherapeutic agents [27]. A study exploring the effects of hypoxia on the expression of P-gp and MDR protein in human lung adenocarcinoma A549 cell line showed that the expression of HIF-1α, P-gp, and MDR protein was higher and the resistance of A549 cells to adriamycin increased under hypoxia [28].

### Hypoxia-mediated regulation of apoptosis

**Fig. 2:**
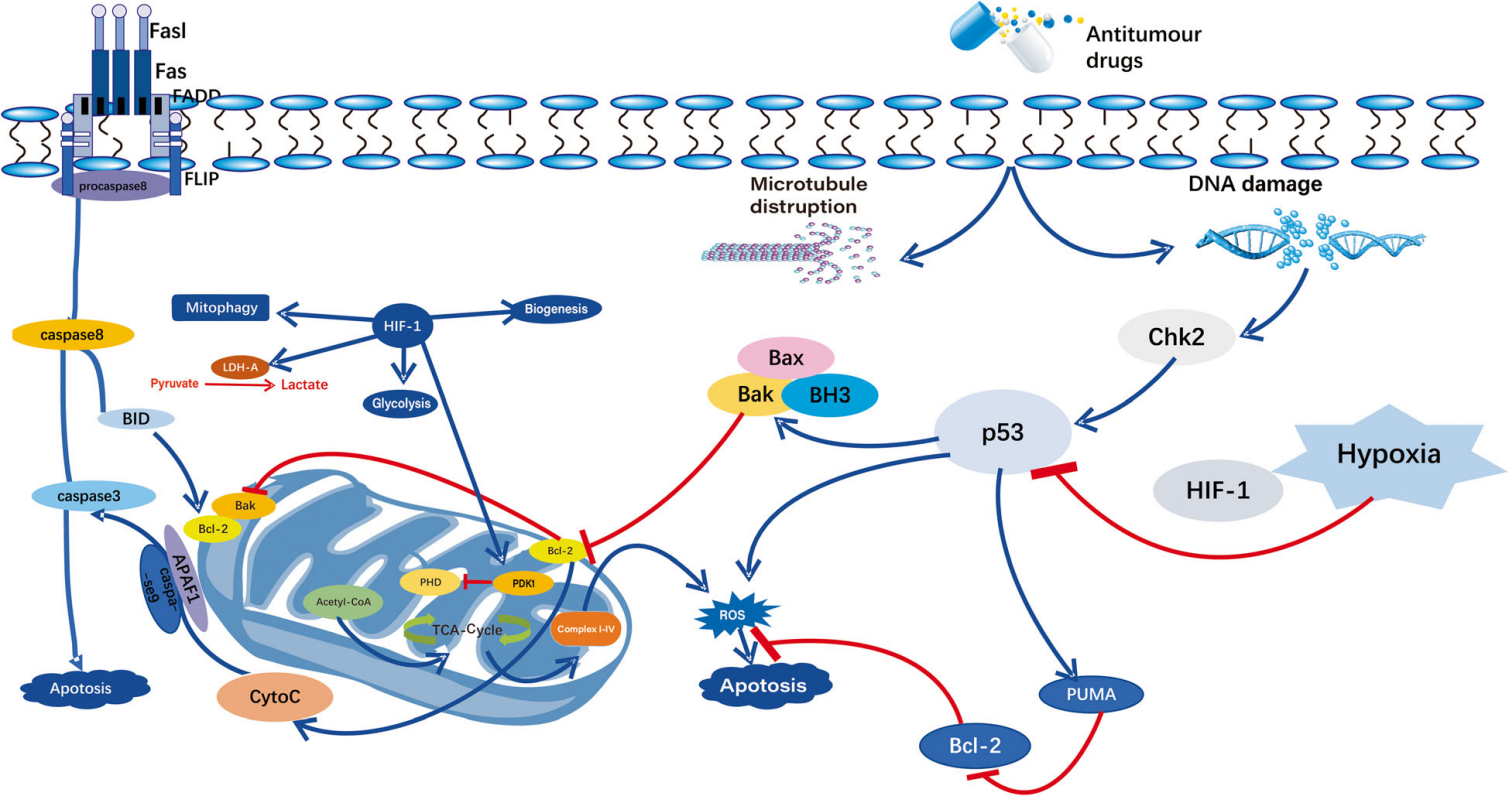
Diagram of the modification effect of HIF-1 on mitochondrial activity. HIF-1 is beneficial for glycolysis and lactic acid production by activating pyruvate dehydrogenase kinase-1 (PDK1) and hindering the activity of pyruvate dehydrogenase (PDH). In addition, HIF-1 targets PDK1, directly inhibiting pyruvate from entering the TCA cycle through the inactivation of PDH. HIF-1 also can induce mitochondrial autophagy and inhibit mitochondrial biogenesis, thus avoiding cell death and ultimately leading to HIF-1-mediated drug resistance

Tumor cells always alter their metabolism to ensure survival and evade host immune attack to proliferate. Defective apoptosis represents another pivotal reason for drug resistance because anticancer treatments act in part by inducing apoptosis, a process mediated by members of the caspase family of proteases (summarized in Fig. 2) [80, 81]. The caspases mediate the selective cleavage of a subset of cellular polypeptides, thereby contributing to the biochemical and morphological features of apoptotic cells [82]. Two main intracellular caspase cascades are triggered by death receptor–ligand systems and various cellular stresses: DNA damage and microtubule disruption. In regulating the activation of these protease cascades, a string of factors, including B-cell lymphoma-2 (Bcl-2) family members, inhibitors of apoptosis-related proteins, and several protein kinases, are closely related [83]. The Bcl-2 protein family impairs the cell’s ability to release apoptogenic protein cytochrome c (cyt c) from the mitochondria by binding to the proapoptotic proteins Bcl-2-associated X protein, apoptosis regulator (Bax), and Bcl-2 homologous antagonist killer, mediating the balance between cell survival and apoptosis [29]. The death-receptor pathway begins with the death-effector domain, which is a critical protein interaction domain recruiting caspases into complexes with the cell surface receptors. Cyt c and other mitochondrial polypeptides were found to be released from the mitochondrial intermembrane space in the mitochondrial pathway [84]. This process involves mitochondrial permeability transition and transfer of certain Bcl-2 family members from the cytoplasm to the outer mitochondrial membranes [85]. The overall survival threshold is probably determined by the balance of interactions between proapoptotic and antiapoptotic members of the Bcl-2 family. Under hypoxic conditions, the nonadaptive cancer cells undergo apoptosis via HIF-1- and P53-dependent mechanisms. The Bcl-2 family can be divided into two categories: anti-apoptotic proteins such as Bcl-2, Bcl-xl, Myeloid cell leukemia-1 (Mcl-1), and cell death abnormality gene 9 (CED9); and pro-apoptotic proteins including mainly BCL2-associated X protein (Bax), BCL2 antagonist/killer (Bak), Bcl-xs, Bad, and Bid. The mRNA and protein levels of proapoptotic Bid and Bad decreased in vitro and in human colon cells with oxygen deprivation. HIF-1 was dispensable for the downregulation of Bad but was required for that of Bid [30]. Antiapoptotic proteins, such as an inhibitor of apoptosis 2 (IAP-2), could be induced whereas the proapoptotic protein Bax could be downregulated in an HIF-1-dependent manner [86]. Abnormalities in the apoptosis machinery lead to a resistant phenotype of tumor cells. During isolation and characterization, several drug-resistant mutants were found to be resistant to antitumor agent–induced apoptosis. For example, a subtractive hybridization of cDNA with mRNA from human monocyte leukemia U937 and its variant UK711 was performed. Glyoxalase I was found to be selectively overexpressed in antiapoptotic UK711 cells [87]. Methylglyoxal is an active dicarboxylic compound involved in a variety of deleterious processes, including the formation of AGE with proteins or DNA modification. In tumor cells, the increased glycolysis activity of the intracellular concentration of methylglyoxal (a byproduct of glycolysis) due to the rapid dysregulation of growth leads to hypoxia in the TME. Methylglyoxal is cytotoxic, and glyoxalase I is an essential component in the detoxification of methylglyoxal. The principal pathway for the catabolism of methylglyoxal is the glyoxalase pathway, which consists of two enzymes, glyoxalase I and glyoxalase II. The mRNA expression and the activity of glyoxalase I in several drug-resistant cells (including UK711, UK110, and K562/ADM) significantly increased compared with parental cells. These mutant cell lines survived after treatment with etoposide or doxorubicin, and the emergence of drug resistance might be accompanied by the overexpression of enzymes. Experiments showed that the overexpression of glyoxalase I in human leukemia cells inhibited etoposide- and doxorubicin-induced apoptosis, indicating that the enzyme was directly involved in the inhibition of apoptosis induced by the chemotherapeutic drug. However, the mechanism by which the overexpression of glyoxalase I inhibits apoptosis has not been fully elucidated. It may be related to the upstream signaling that inhibits apoptosis, leading to the activation of caspase [88, 89]. HIF-1 protected against DNA-damage-induced germ cell apoptosis by antagonizing the function of CEP-1, the homolog of the tumor suppressor p53 [89, 90]. Further, p53^+/+^ cells exposed to hypoxia exhibited a transient arrest in G2/M compared with 43 isogenic p53-null cells exposed to hypoxic conditions, exhibiting a sixfold to tenfold higher level of apoptosis. Hence, it was hypothesized that p53 acted as a survival factor under limiting oxygen concentrations [91]. Thus, an understanding of cell death processes after cytotoxic damage might help in cancer treatment.

### Hypoxia-mediated induction of autophagy

Besides apoptosis, the process of autophagy is widely recognized as a critical regulator of cellular viability [92]. Autophagy means self-digestion. It is a catabolic process of phagocytizing cytoplasmic proteins or organelles to degrade the contents of cells, thereby realizing the metabolism of cells themselves [93]. Autophagy in tumor cells is a double-edged sword. On the one hand, autophagy can remove misfolded proteins and dysfunctional organelles within tumor cells, such as mitochondria, inhibit cellular stress response, and ultimately prevent genomic damage, thereby inhibiting cancer. On the other hand, in the advanced stage of tumor growth, tumor cells can make use of autophagy to survive in the condition of nutrient deficiency or hypoxia [94]. Although the precise role of autophagy in tumor formation is controversial, the state of autophagy is closely associated with the failure of antiproliferative treatment in neoplastic cells [95]. Currently, three autophagic mechanisms have been identified: macroautophagy, microautophagy, and chaperone-mediated autophagy (CMA). Autophagy-associated (ATG) proteins, upon the receipt of autophagy signals associated with autophagosome formation, trigger the activation of macroautophagy. The initiation of autophagosome formation is governed by the complex ULK-1–Atg13–FIP200, which is negatively regulated by the mammalian target of rapamycin (mTOR) by phosphorylating ULK [96]. The LKB1–AMPK axis (an energy sensor) is activated by energy starvation through phosphorylation, directly initiating ULK1–Atg13–FIP200 complex formation, or through TSC1/2-dependent or -independent mTOR inhibition. The functional inactivation of autophagy pathways results in significantly enhanced efficacy of chemotherapeutic agents [32]. Autophagy induced by hypoxia is primarily located in hypoxic tumor regions. The link between unfolded protein response (UPR) and the regulator of autophagy involving PERK-dependent transcriptional induction of MAP 1LC3B and ATG has also been identified. The data showed that UPR was a key mediator in promoting therapy resistance through its ability to facilitate autophagy [97]. Substantial evidence has shown that autophagy promotes the development of MDR. Since ABC transporters are associated with MDR, agents that modulate ABC transporters have been advocated as chemotherapeutic drugs to overcome MDR. However, the current clinical efficacy of ABC transporter modulators has not achieved the desired results. MDR is an extremely complex phenotype. An increasing number of studies have focused on clarifying the link between autophagy and MDR based on clinical data. The elevated levels of autophagy detected in patients with poor prognosis indicate that autophagy can catalyze the development of MDR. Doxorubicin and vincristine treatment upregulates the expression of S100A8, which is required for the formation of the Beclin1–III phosphatidylinositol 3-kinase (PI3KC3) complex [98]. MDR can be mediated by high-mobility box group 1 (HMGB1), which promotes protective autophagy against anticancer agents. HMGB1 is transferred from the nucleus to the cytoplasm and promotes the formation of the Beclin1–PI3KC3 complex by activating the mitogen-activated protein kinase (MAPK)/extracellular signal-regulated kinase (ERK) signaling pathway [99]. Likewise, peptidylarginine deiminase IV can cause MDR in hepatocellular carcinoma cells by inducing autophagy as a protective function [100]. MiR-23b-3p inhibited autophagy mediated by ATG12 and HMGB2 and restored the sensitivity of MDR cells to chemotherapy in vivo [33]. Cumulative evidence suggested that autophagy, as a cytoprotective mechanism, mediated MDR, thereby protecting MDR cancer cells from apoptosis and promoting resistance to chemotherapy treatment. However, undoubtedly, the cells are autophagy dependent under the stress of nutrient deficiency. Chemotherapeutic agents known to cause DNA damage (temozolomide and cisplatin) or inhibition of DNA synthesis [5-fluorouracil (FU) and gemcitabine] and other agents that target signal transduction pathways due to specific gene mutation, amplification, and activation, such as erlotinib and gefitinib (EGFR mutation), imatinib (tyrosine kinase activation), and trastuzumab (HER2 amplification), induce growth inhibition and lead to autophagy-mediated cell survival. These findings indicated that autophagy inhibitors, such as chloroquine (CQ) and hydroxychloroquine (HCQ), against autolysosome formation combined with the aforementioned therapeutic agents could abrogate autophagy-dependent cell survival [34, 101–104].

### Hypoxia-mediated inhibition of DNA damage

Most anticancer drugs kill tumor cells by causing DNA damage, which is considered as the basic mode of action for a majority of classical chemotherapeutic agents. However, cancer cells can activate various distinct repair mechanisms and signaling pathways as a response to overcome DNA damage. Subsequently, repaired cancer cells become more resistant to chemotherapeutic treatment [105, 106]. On exposure to hypoxia, cancer cells undergo replication stress, thereby activating DNA damage and repair pathways. Two main kinase signaling cascades exist: (1) ATM–Chk2 and (2) ATR–Chk1 pathways activated to induce cell cycle arrest. For example, the E3 ubiquitin ligase Smurf1 determines cell apoptosis rates downstream of DNA damage–induced ATR–Chk1 signaling by promoting the death of transformed cells [107, 108]. HIF-1α is associated with the increased chemoresistant phenotype of cancer cells, and downregulated HIF-1α can increase the sensitivity toward etoposide treatment. In an HIF-1α-deficient cell model, the transcript levels of DNA–PK complex members, DNA–PKcs and Ku80, were downregulated, leading to a higher susceptibility to chemotherapeutics and introducing DNA double-strand breaks [35]. The analysis of breast (MDA-MB-231) and liver (HepG2) cancer cell lines treated with taxol or etoposide under hypoxic conditions revealed that hypoxic cells showed less sensitivity to drug treatment compared with normoxic cells. The transcriptome analysis showed that the expression of transmembrane protein 45A increased in hypoxic cells, inducing cell cycle arrest and resistance to etoposide-induced DNA strand breaks [36]. In addition, the role of p53 in hypoxic drug resistance is indispensable. In cancer cells, hypoxia disrupts the p53–RPA70 complex, which in turn enhances RPA70-mediated nucleotide excision repair/nonhomologous end-joining repair whereas RPA70 binds to the p53-N-terminal domain in normal cells. Under the hypoxic stress, cancer cells activate the phosphorylation of p53 in the serine 15 region, thereby inhibiting the formation of p53–RPA70 complex [104]. However, a number of unresolved issues need to be considered when establishing a link between hypoxia and genetic instability and invasive phenotypes. Whether DNA repair defects caused by hypoxia in precancerous models and permanent alteration of genomic stability and cell transformation, rather than transient hypoxia, alter protein expression to drive cell proliferation, survival, and metastasis phenotype needs confirmation. These concepts are effectively translated into clinical needs to quantify and monitor acute and chronic hypoxia, thus providing options for individualized cancer treatment. The present model did not distinguish between the complexity of the hypoxic microenvironment and the biological effects of acute and chronic hypoxia–mediated transcriptional and translational changes. Hence, it is necessary to identify molecularly targeted drugs under different conditions, including normal oxygen levels and hypoxia (acute and chronic), equally inhibiting the targeting molecule or the pathway, helping to ensure that clinical resistance is not just due to drugs ineffective in hypoxic cells. Also, whether cancer stem cells preferentially exist in tumor hypoxic regions needs confirmation [109].

### Hypoxia-mediated downregulation of mitochondrial activity

In the process of converting glucose into lactate that maintains the balance of redox homeostasis and cell survival under hypoxic conditions, HIF-1 plays a key role in the reprogramming of cancer metabolism (summarized in Fig. 2) [110]. The increase in glycolysis for ATP generation in cancer cells is frequently associated with resistance to therapeutic agents. Cells incubated under hypoxic conditions produced a significantly larger amount of lactate than those under normoxic conditions along with reduced sensitivity to doxorubicin and ara-c in both human leukemia cell line HL-60 (HCT116 cells) and human lymphoma cell line Raji (Raji cells) [111]. However, cancer cells less sensitive to chemotherapeutic agents could be effectively killed using glycolytic inhibitors. Even cells that expressed an MDR phenotype still remained sensitive to the inhibition of glycolysis. Mitochondria are indispensable for the nonapoptotic forms of cell death. Hence, the mechanism underlying the involvement of HIF in altering mitochondrial function in cancer needs to be explored [112]. In mammalian cells, HIF-1 can regulate the expression of cyt c oxidase–cytochrome c oxidase subunit IV (COX4) by activating the transcription of genes encoding COX4–2 and LON to optimize the efficiency of respiration under hypoxic conditions [113]. HIF-1 stimulates glycolysis, and the TCA cycle is suppressed by the molecular mechanism inducing PDK1 [114]. HIF-1 is required for tumorigenesis in VHL-deficient renal carcinoma cells, and these effects are mediated by HIF-1 via inhibiting C-MYC activity, which determines the transcription of the gene encoding the coactivator PGC-1b [37, 115]. The loss of PDG-1b contributes to decreased respiration in VHL-deficient renal carcinoma cells. Mitochondria and HIFs are closely connected to regulate each other during the cellular death pathways. The suppression of mitochondrial activity qualifies as a reliable mechanism of HIF-driven treatment failure [38]. Otherwise, not only drug and radiation can induce ROS production for killing tumor cells, but also mitochondria can induce death signaling via the generation of ROS. Evidence showed that cells depleted of their mitochondrial DNA were not able to elicit hypoxia-induced increase in ROS generation and HIF-1α-induced ROS protein accumulation. In human oral squamous cell carcinoma cell (OSCC) lines, the forced expression of HIF-1α suppressed the generation of ROS and increased cytosolic accumulation of cyt c, inhibiting the hypoxia-induced apoptosis of OSCC lines [39]. The model proposed for the HIF-1-dependent regulation of chemosensitivity by ROS and the experimental result showed that HIF-1-competent cells displayed a more chemoresistant phenotype. Given the central role of mitochondria, the comprehensive connection between HIF, mitochondrial activity, and chemoresistance provides a new direction of research for explaining therapy failure and proposing innovative treatment strategies [116].

### Hypoxia-mediated regulation of p53

The TP53 gene is by far the most frequently mutated gene in human cancer [117, 118]. P53-deficient mice are often associated with a high incidence of spontaneous tumors, indicating strong TP53 inactivation during tumor development. HIF-1α has been proved to regulate p53 tumor suppressor protein levels to adapt to hypoxia. Conversely, p53 can also regulate HIF-1α protein levels negatively. Under mild hypoxic conditions, p53 protein levels are decreased to protect cells against apoptosis and promote cell survival. However, under severe hypoxic, even anoxic, conditions, p53 protein levels are stabilized, thereby decreasing HIF-1α transcriptional activity and inducing apoptotic cell death [119]. The HIF-1-mediated transcriptional upregulation of the secreted tyrosinase (TYR) TYR-2 (or the human homolog TRP2) was identified as the underlying p53-inhibiting mechanism. The activation of the p53 pathway upon treatment with chemotherapeutic agents was found to be markedly suppressed. Moreover, the accumulation level and activity of HIF-1α increased in p53 mutant cells, thereby allowing decreased apoptotic potential and chemoresistant properties [120, 121]. Resistance to cisplatin frequently occurs in non-small-cell lung cancer (NSCLC). Christoph et al. provided evidence that this sensitivity to cisplatin could be reversed after reoxygenation in a time-dependent manner in A549 NSCLC cells [40]. By binding with the DNA helicase Xeroderma pigmentosum group B (XPB), active p53 allows nucleotide excision repair and protects cells from chemotherapy-induced apoptosis. Studies found that in colorectal cancer (CRC) cell lines, prolyl hydroxylase domain protein PHD1 silencing could hinder p53 activation on chemotherapy treatment and reduce DNA repair to favor cell death. The simultaneous observation was that *PHD1* silencing sensitized the response of CRC to 5-FU in mice [122]. Such a new posttranslational modification in the p53 field may be used in combination with chemotherapy to increase sensitivity to treatments.

### Hypoxia-mediated reduced efficiency in chemotherapy

Chemotherapy drugs are still the cornerstone of cancer treatment. They have an oxygen-dependent effect on the killing of tumor cells, most of which kill cells by oxidizing free radicals and ROS in cells. The antibiotic bleomycin has a reduced efficiency in chemotherapy under hypoxic conditions, which may be related to the reduction of free radicals. Triche et al. showed that platinum-based chemotherapeutic drugs generated free radicals in cells, which captured electrons and delivered them to oxygen, thereby killing cells [123]. Wozniak et al. found that etoposide inhibited DNA strands more frequently under hypoxic conditions than under normoxic conditions due to increased levels of free radical scavenger dehydrogenase inhibitors and dehydrogenase substrates [124]. The aforementioned studies suggested that hypoxia had a significant inhibitory effect on the efficiency of chemotherapy. Hence, cancer resistance could be overcome by accurately interfering with hypoxia to improve the therapeutic effect.

## Targeting hypoxia and HIFs in cancer

Hypoxia is arguably one of the most attractive therapeutic targets in cancer. Several approaches for targeting hypoxic tumor cells have been proposed, including hypoxia-activated prodrugs, gene therapy and specific targeting of HIFs, or targeting pathways important in hypoxic cells such as mTOR and UPR pathways. Otherwise, this feature can be used to target acid-induced tumors because tumor tissues have lower pH values compared with normal tissues.

A prodrug is an inactive compound that can be converted into pharmacologically active substances spontaneously or through specific metabolic pathways [125]. An anoxic prodrug activated in anoxic tissues was designed to kill anoxic tumor cells selectively using the characteristics of anoxic tumors. Hypoxic prodrugs are activated by cellular reductases, reoxidized into initial drug progenitors in anoxic cells, and converted into cytotoxic substances. The results of the recently published phase II clinical trial on the hypoxic progenitor TH-302 combined with gemcitabine for pancreatic cancer or adriamycin for soft tissue sarcoma are encouraging [126]. The efficacy of another mitomycin C derivative prodrug praziquantel (EO9) has been shown in preclinical studies. Therefore, topical praziquantel is recommended as adjunctive therapy for patients undergoing bladder cancer surgery [127].

Another strategy is to target and regulate HIF-1α in solid tumors to overcome resistance to hypoxia. For example, strategies for hypoxia can be targeted at downstream HIF signaling pathways. Monoclonal antibodies that target VEGF (bevacizumab) or small-molecule inhibitors that target VEGF receptors have achieved clinical benefits for advanced cancer. Methods for inhibiting the HIF response to hypoxia include siRNA treatment, blocking the dimerization of HIF-1α and β subunits, and direct inhibition of HIF-1α using anticancer agents known to inhibit the PI3K/AKT/HIF-1α pathway. Furthermore, drugs that trigger the activation of HIF-1α degradation pathways have the potential to clear overexpressed HIF-1α in hypoxic tumors. Treatments of hypoxic cells with rapamycin lead to the degradation of HIF-1α, which results in the increased inhibition of the expression of survivin and apoptosis in lung cancer cells. In addition, Shukla et al. found that HIF-1α mediated the resistance of pancreatic cancer cells to gemcitabine by upregulating the expression of cytidine triphosphate synthase (CTPS1) and transketolase (TKT). When digoxigenin is used to inhibit the translation of the HIF-1α subunit, pancreatic cancer cells are more sensitive to gemcitabine [128]. Potential molecular therapeutic targets based on the TME and breast cancer mechanism have been widely studied. Studies have shown that HIF-1α induces P4HA1 expression in a hypoxic microenvironment, and P4H can regulate cell metabolism and enhance the activity of tumor cells. Hence, targeting P4H is a potential strategy to improve the treatment of breast cancer [129]. Cui et al. found that HIF-1α was involved in the transcriptional regulation of the *TFPI* gene, and the hypoxic microenvironment in breast tumors can induce the procoagulant status in patients with breast cancer [130]. HIF-1α may be a target for the treatment of breast cancer–related coagulation and thrombosis. The oxygen sensor hypoxia-inducible factor prolyl hydroxylase 2 (PHD2) is considered to be the major regulator of HIF-1α. Kozlova et al. described a significant positive correlation between PHD2 and EGFR expression and introduced hypoxia/PHD2-mediated signaling and EGFR-induced tumor mitigation in patients with breast cancer [131]. This is of profound significance for the targeted treatment of breast cancer.

As illustrated earlier, cancer cells have higher levels of ROS and overexpressed antioxidant enzymes. Therefore, the elimination of enzymes involved in antioxidant defense results in higher oxidative stress, thus leading to the death of resistant cells. ROS modulators are effective in overcoming MDR in cancer cells through this principle of action. Moreover, target acid–induced tumors can improve the specificity of tumor therapy. In recent years, the application of proton pump inhibitors (PPIs) in treating the acidic environment is popular. Studies have shown that PPIs can increase the uptake of cisplatin cells in an acid-dependent manner and enhance the role of cytotoxic agents in chemotherapy-resistant epithelial ovarian cancer [89, 132]. Intervention strategies targeting the acidic microenvironment and novel combination therapy strategies may be future research directions.

## Future perspectives

Drug resistance is a complex multifactorial phenomenon. Despite significant advancements in cancer care, especially the development of targeted anticancer therapies, the mechanisms of protecting cells from cytotoxic compounds mediated by tumor–host interactions continue to play a primary role as obstacles to cancer therapy. Evidence shows that these mechanisms determine the sensitivity to cancer treatment. Chemotherapy drugs exert an influence on biological damage by activating diverse signaling pathways of the cell death program. Defects in cell death processes controlling apoptosis (such as tolerating DNA damage) or promoting survival (such as efficient repair) lead to insensitivity to antitumor agents. In addition, in vitro toxicity screening performed at the standard air pressure is of paramount importance, but fails to recognize the impact of the TME. Fortunately, it is now widely acknowledged that hypoxia is responsible not only for pharmacokinetics but also for supporting the selection of more malignant cells or even resistance. Many cancer research laboratories are actively involved in the identification of novel therapies to target hypoxia. According to the characteristics of HIF in tumor biological behavior, the therapeutic strategies can be optimized by combining with the molecular mechanisms that drive therapy resistance, thus helping in the progress of cancer care. Therefore, a detailed understanding of the regulation of apoptosis, growth inhibition, and DNA repair, besides the identification of defects in the death pathway, may provide insights into the mechanisms of clinically relevant drug resistance. Targeting hypoxia is a potential therapy to eradicate the progression of various cancers and enable long-term survival for patients.

## Conclusions

Clinical studies have demonstrated that the components in the tumor hypoxic microenvironment are associated with poor prognosis in patients and can promote apoptosis and autophagy or inhibit DNA damage and mitochondrial activity through a number of signaling pathways associated with the failure of immunotherapy, chemotherapy, or radiation therapy. Especially evident in advanced metastatic cancer, a hypoxic environment is often established, which plays an important role in cancer evolution. Further investigations proved that HIF-1α was involved in hypoxia-induced therapy resistance, and its knockdown could reverse the resistance. Hence, it was inferred that the selection pressure of tumor hypoxic environment ecology could affect the evolution of cancer cells. HIF-1 facilitates glycolysis and lactate production through the key enzymes of glycolysis and LDH-A, thereby inhibiting the entry of pyruvate into the TCA cycle. Furthermore, the HIF-1 target gene *PDK1* directly inhibits the migration of pyruvate to mitochondria by inactivating pyruvate dehydrogenase (PDH). An increasing number of studies have shown that HIF-1 can induce mitochondrial autophagy and inhibit mitochondrial biosynthesis to inhibit cell death, ultimately leading to HIF-1-mediated resistance. The hypoxia-induced acidic microenvironment of tumor is very important for chemoresistance, in some cases promoting EMT and stem cell–like phenotypes. Major factors such as V-ATPase, NHE, and MCT in regulating the tumor acidic microenvironment also provide targets for tumor therapy. Therefore, understanding the regulation of these molecules is important for identifying potential therapeutic targets. A better understanding of the pathways in the hypoxic environment during tumor progression may contribute to breakthroughs in cancer immunotherapy research and provide a theoretical basis for clinical trials to help improve treatment outcomes.

## Data Availability

The material supporting the conclusion of this study has been included in the manuscript.

## Abbreviations

ABC: ATP-binding cassette
Bak: BCL2 antagonist/killer
BAX: Bcl-2-associated X protein
Bcl-2: B-cell lymphoma-2
bHLH: Basic helix-loop-helix
CBP: CREB-binding protein
CHK2: Checkpoint kinase 2
CMA: Chaperone-mediated autophagy
COX4: Cytochrome c oxidase subunit IV
CQ: Chloroquine
CRC: Colorectal cancer
Cyt c: Cytochrome c
DED: Death-effector domain
DSBs: Double-strand break
EM: Estramustine
FCM: Flow cytometry
HCQ: Hydroxychloroquine
HIF-1α: Hypoxia-inducible factor 1α
HIFs: Hypoxia-inducible factors
LDH-A: Lactate dehydrogenase-A
Mcl-1: Myeloid cell leukemia-1
MRP1: Multidrug resistance protein 1
mTOR: Mammalian target of rapamycin
NER: Nucleotide excision repair
NHEJ: Nonhomologous end-joining
Nrf2: Nuclear factor (erythroid-derived 2)-like factor 2
NSCLC: Non-small-cell lung cancer
NTD: N-terminal domain
OSCC: Oral squamous cell carcinoma cell
PAS: Per-ARNT-Sim
PDK1: Pyruvate dehydrogenase kinase1
P-gp: P-glycoprotein
PHD: Prolyl hydroxylase domain
ROS: Reactive oxygen species
TCA: Tricarboxylic acid cycle
TME: Tumor microenvironment
TMEM45A: Transmembrane protein 45A
TYR-2: Tyrosinase-2
ULK: Unc-51-likekinase
UPR: Unfolded protein response
VEGF: Vascular endothelial growth factor
VHL: Von Hippel–Lindau
XPB: Xeroderma pigmentosum group B
